# Impact of Birth Weight and Early Infant Weight Gain on Insulin Resistance and Associated Cardiovascular Risk Factors in Adolescence

**DOI:** 10.1371/journal.pone.0020595

**Published:** 2011-06-02

**Authors:** Signe Fabricius-Bjerre, Rikke Beck Jensen, Kristine Færch, Torben Larsen, Christian Mølgaard, Kim Fleischer Michaelsen, Allan Vaag, Gorm Greisen

**Affiliations:** 1 University Department of Neonatology, Rigshospitalet, Copenhagen, Denmark; 2 University Department of Growth and Reproduction, Rigshospitalet, Copenhagen, Denmark; 3 Steno Diabetes Center, Gentofte, Denmark; 4 Department of Obstetrics and Gynaecology, Sygehus Nord, Holbæk Hospital, Holbæk, Denmark; 5 Department of Human Nutrition, Faculty of Life Sciences, University of Copenhagen, Frederiksberg, Denmark; Cardiff University, United Kingdom

## Abstract

**Background:**

Low birth weight followed by accelerated weight gain during early childhood has been associated with adverse metabolic and cardiovascular outcomes later in life. The aim of this study was to examine the impact of early infant weight gain on glucose metabolism and cardiovascular risk factors in adolescence and to study if the effect differed between adolescents born small for gestational age (SGA) vs. appropriate for gestational age (AGA).

**Methodology/Principal Findings:**

Data from 30 SGA and 57 AGA healthy young Danish adolescents were analysed. They had a mean age of 17.6 years and all were born at term. Data on early infant weight gain from birth to three months as well as from birth to one year were available in the majority of subjects. In adolescence, glucose metabolism was assessed by a simplified intravenous glucose tolerance test and body composition was assessed by dual-energy X-ray absorptiometry. Blood pressures as well as plasma concentrations of triglycerides and cholesterol were measured. Early infant weight gain from birth to three months was positively associated with the fasting insulin concentration, HOMA-IR, basal lipid levels and systolic blood pressure at 17 years. There was a differential effect of postnatal weight gain on HOMA-IR in AGA and SGA participants (*P* for interaction = 0.03). No significant associations were seen between postnatal weight gain and body composition or parameters of glucose metabolism assessed by the simplified intravenous glucose tolerance test. In subgroup analysis, all associations with early infant weight gain were absent in the AGA group, but the associations with basal insulin and HOMA-IR were still present in the SGA group.

**Conclusion:**

This study suggests that accelerated growth during the first three months of life may confer an increased risk of later metabolic disturbances – particularly of glucose metabolism – in individuals born SGA.

## Introduction

Small size at birth is associated with a range of metabolic diseases in adulthood, including type 2 diabetes, hypertension, and dyslipidemia [1], [2]. However, controversy exists as to whether accelerated growth in infancy is disadvantageous. Some studies have shown that rapid weight gain in the early postnatal period has adverse consequences for later health [3]–[6]. Others have reported that low weight at one year of age or low weight gain in the first year of life – in some cases followed by accelerated growth during childhood – can contribute independently to the risk of developing metabolic and cardiovascular disease later in life [7]–[11]. Accordingly, different pathways of early growth seem to precede the development of metabolic disturbances later in life [12], [13].

The effect of postnatal growth on later health and disease depends to a high extent on birth weight. Indeed, rapid postnatal growth may have adverse effects on later health particularly in individuals born small for gestational age (SGA)[3], [14]–[17] (i.e. catch-up growth). Thus, a potential confounder in some of the studies finding adverse effects of accelerated growth in infancy may be a lack of correction for catch-up growth elicited by low birth weight *per se*. Catch-up growth may be due to the fetus having escaped an adverse intrauterine environment and may represent a major adaptive mechanism with independent late consequences [13]. However, catch-up growth can also reflect a more trivial result of the general statistical phenomenon ‘regression towards the mean’.

We have previously shown that low birth weight for gestational age, but not restricted fetal growth in the 3^rd^ trimester, was associated with insulin resistance and cardiovascular risk factors in adolescence [18]. Accordingly, the impact of low birth weight on metabolic disturbances in adolescence do not seem to be due to restricted intrauterine growth, but may be related either to factors operating prior to the third trimester or to postnatal catch-up growth. The aim of the current study was to examine the impact of early infant weight gain (from birth to 3 and 12 months) on health in adolescents born small for gestational age (SGA) and appropriate for gestational age (AGA).

## Methods

### Ethics statement

This study was performed according to the Helsinki II declaration and was approved by the local ethics committee for Copenhagen and Frederiksberg (KF 01-229/02 & KF 01-065/03). Informed consent was obtained from all participants and from the parents when the participant was under 18 years of age.

### Cohort study of fetal and postnatal growth (1985–1987)

In a randomised controlled trial performed in 1985 to 1987 [19], data were collected on third trimester fetal growth velocity, birth weight for gestational age and early infant growth in the first year of life. Birth weight for gestational age and intrauterine growth velocity were used as inclusion criteria for the follow-up at adolescence, and postnatal growth data were used for the present analyses.

**Figure 1:**
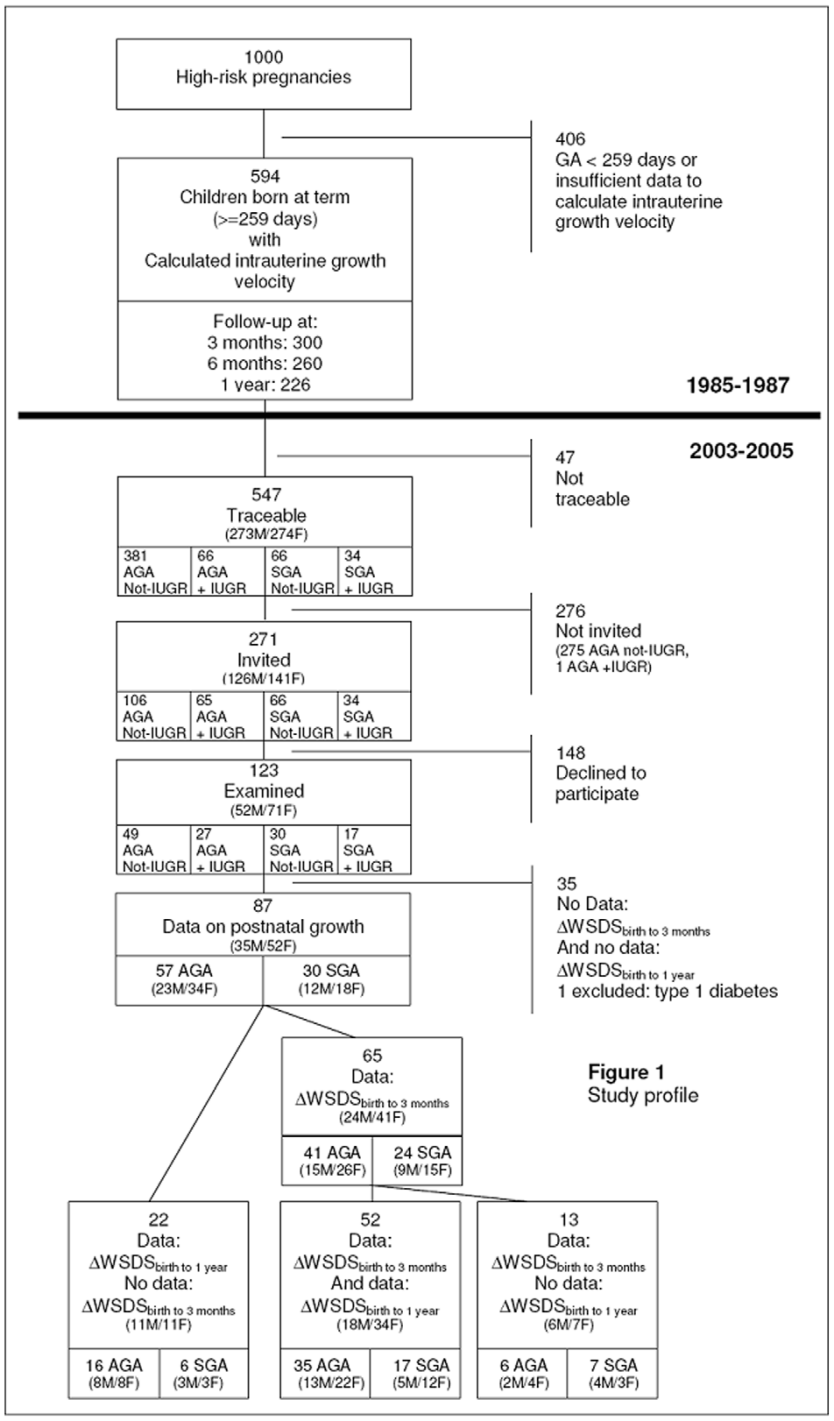
Study design. AGA: Appropriate for gestational age. GA: Gestational age. SGA: Small for gestational age. IUGR: Intrauterine growth restricted. ΔWSDS_birth to 3 months_: Infant weight gain from birth to 3 months. ΔWSDS_birth to 1 year_: Infant weight gain from birth to 1 year.

The study design is shown in Figure 1. One thousand pregnant women were included in the original trial on the basis of one or more risk factors for giving birth to a SGA child. The list of risk factors has been reported in detail previously [18]. Ultrasound at 18 weeks was used to establish gestational age, and ultrasound from 28 weeks was used to estimate fetal weight repeatedly [20]. Fetal growth velocity in the third trimester (FGV) could be determined in 594 subjects born at term, and was expressed as change in standard deviation scores (SDS) of fetal weight per 28 days [21] using a sex-specific reference of fetal growth [22]. Birth weight SDS was calculated using the same growth reference [22]. Intrauterine growth restriction (IUGR) was defined as declining growth in the third trimester using the cut-off of FGV below the 10^th^ percentile (−0.39 SDS/28 days). SGA was defined as birth weight SDS below the 10^th^ percentile (-1.28 SDS), and AGA was defined as birth weight SDS above this cut-off. The relationships of FGV and IUGR with insulin resistance and associated cardiovascular risk factors in adolescence have been published elsewhere [18].

Follow-up examinations were performed at 3, 6 and 12 months on half of the cohort [21]. Weight, length, and head circumference were measured and transformed to SDS using a national chart of infant growth [23].

### Follow-up in adolescence 2003–2005

A total of 547 offspring were traceable, and among these 271 were invited to participate (all those who were SGA or IUGR and a random sample of AGA/not-IUGR) (Figure 1). The study was predicted to have the power of 80% to detect a difference in mean arterial blood pressure of 5 mmHg. One hundred and twenty three subjects (52 male, 71 female) agreed to participate. Participants and non-participants did not differ statistically significant concerning birth weight SDS (−0.63±1.21 vs. −0.61±1.11 SDS)(P = 0.88) or FGV (−0.17±0.40 vs. −0.19±0.38 SDS/28 days)(P = 0.78). Supplementary data (*n* = 27) for the calculation of infant weight gain from birth to one year was collected by questionnaire. In total, 87 individuals (40% males) had data on weight SDS at three months and/or at one year and were included in the analyses. Of these, 57 were AGA and 30 were SGA (Figure 1, Table 1). The gender distribution did not differ significantly between the SGA and AGA groups (*P* = 0.574). There were no statistically significant differences in the measured outcomes at follow-up between individuals with postnatal growth data and those without.

**Table 1:** Anthropometry at birth, infancy and adolescence of subjects born appropriate for gestational age (AGA) and small for gestational age (SGA).

	AGA (n = 57)	SGA (n = 30)
Males (%)	40	40
**Birth**		
Birth weight (g)	3516±409	2714±284***
Birth weight SDS	0.17±0.86	−1.81±0.42***
Gestational age (days)	278±8.8	277±9.1
**Infancy**		
ΔWSDS_birth to 3 months_	−0.25±0.76	0.78±0.59***
ΔWSDS_birth to 1 year_	−0.71±1.11	0.54±0.79***
**Adolescence**		
Age at examination (years)	17.5±0.7	17.9±0.7*
Height (cm)	172.5±7.9	167.0±8.7*
BMI (kg/m^2^)	22.9±4.0	22.2±4.4
Waist-hip ratio	0.79±0.05	0.81±0.10
Trunk fat (%)	34 [24 to 47]	35 [26 to 48]

Data are means ± SD or [95% confidence limits] in the AGA and SGA group for the 87 subjects with data on early infant weight gain. ΔWSDS  =  change in weight SDS. ***P<0.001, *P<0.05.

Validated questionnaires [24]–[26] were used to assess lifestyle factors of the participants and their parents, as well as breastfeeding (only breastfeeding exceeding three months was considered in these analyses).

One subject (AGA) was excluded from all analyses because of a diagnosis of type 1 diabetes. Polycystic ovary syndrome had previously been diagnosed in one girl; she was included in the analyses; exclusion did not alter the results.

### Experimental protocol

The experimental protocol has been described in detail previously [18]. In brief, it included fasting blood sampling, a simplified intravenous glucose tolerance test (IVGTT) [27], as well as measures of height (to the nearest 0.1 cm), weight, waist and hip circumference, fat mass and lean mass by dual x-ray absorptiometry (DXA) and resting systolic and diastolic blood pressure (BP). Trunk fat was expressed in percentage of total fat mass.

Female participants who did not use oral contraceptives (n = 29 of 71) were examined at day 2 to 5 of their menstrual period.

### Biochemical analyses

Blood samples for measurement of plasma insulin were centrifuged immediately at 4°C and stored at −20°C until plasma insulin concentrations were determined by the AutoDELPHIA Time-resolved Fluoroimmunoassay (Perkin Elmer-Wallac Oy, Turku, Finland) (total interassay CV<6%). Plasma glucose concentration was measured using the Hexinase/G6P-DH-Determination (Hitachi 912 System; Roche Diagnostics, Hvidovre, Denmark) (intra-assay precision: SD = 0.09 mmol/l; interassay precision: SD = 0.15 mmol/l). Plasma triacylglycerol and cholesterol (total, HDL-cholesterol and LDL-cholesterol) were measured using an enzymatic absorption photometric method (Roche Modular Analytics [SWA] P-module; Roche Diagnostics) (CV<5%).

### Calculations

Infant weight gain from birth to three months (ΔWSDS_birth to 3 months_) was calculated as ((weight SDS_3 months_ – birth weight SDS)/ (age at measurement))*91 days. Infant weight gain from birth to one year (ΔWSDS_birth to 1 year_) was calculated as: ((weight SDS_one year_ – birth weight SDS)/(age at measurement)*365 days. For those who were not measured at one year in the original trial, we used a measurement of weight between 11 and 13 months (both months included), if reported in the questionnaire.

According to the methods described by Galvin *et al.* [27], and using the trapezoidal rule, we calculated: first-phase insulin response (FPIR) as Δ insulin area above basal_0–10 min_ divided by Δ glucose peak above basal_0–10 min_, glucose disappearance rate (*K*_g_) (slope of log glucose concentration_10–40 min_), and insulin sensitivity (SI) (*K*_g_/Δ insulin area above basal_0–40 min_). Disposition index (DI), representing insulin secretion corrected for the ambient degree of insulin resistance, was also calculated (SI×Δ insulin area_0–10min_). Furthermore, mean basal glucose (mean of three basal samples), mean basal insulin (mean of three basal samples) and homeostasis model assessment of insulin resistance (HOMA-IR) were calculated [28].

### Statistical analyses

Dependent variables that did not fulfil the assumption of normality were logarithmically transformed, which in all cases gave distributions close to normal. Effects of ΔWSDS_birth to 3 months_ and ΔWSDS_birth to 1 year_ on later health were analysed separately. All multiple linear regression analyses were adjusted for sex. Effects of post-natal growth on fat and lean body mass were adjusted for current weight, and effects of post-natal growth on blood pressure were adjusted for height and age. Age at examination and the use of oral contraceptives were not included in the analyses, because preliminary analyses showed that inclusion of these variables did not alter the results. Further adjustments for current BMI, fat mass and fat percentage were performed, each in separate analyses. Using general linear models with interaction terms between early infant weight gain and AGA/SGA status, we determined whether the effects of early infant weight gain on later health differed significantly between the SGA and AGA groups. Individuals born AGA and SGA were compared to each other using Students t-test for the continuous variables and Chi^2^-test for frequencies. All statistical analyses were performed using the statistical package SPSS (version 14; SPSS, Inc., Chicago, IL).

## Results

### Subject characteristics

**Figure 2:**
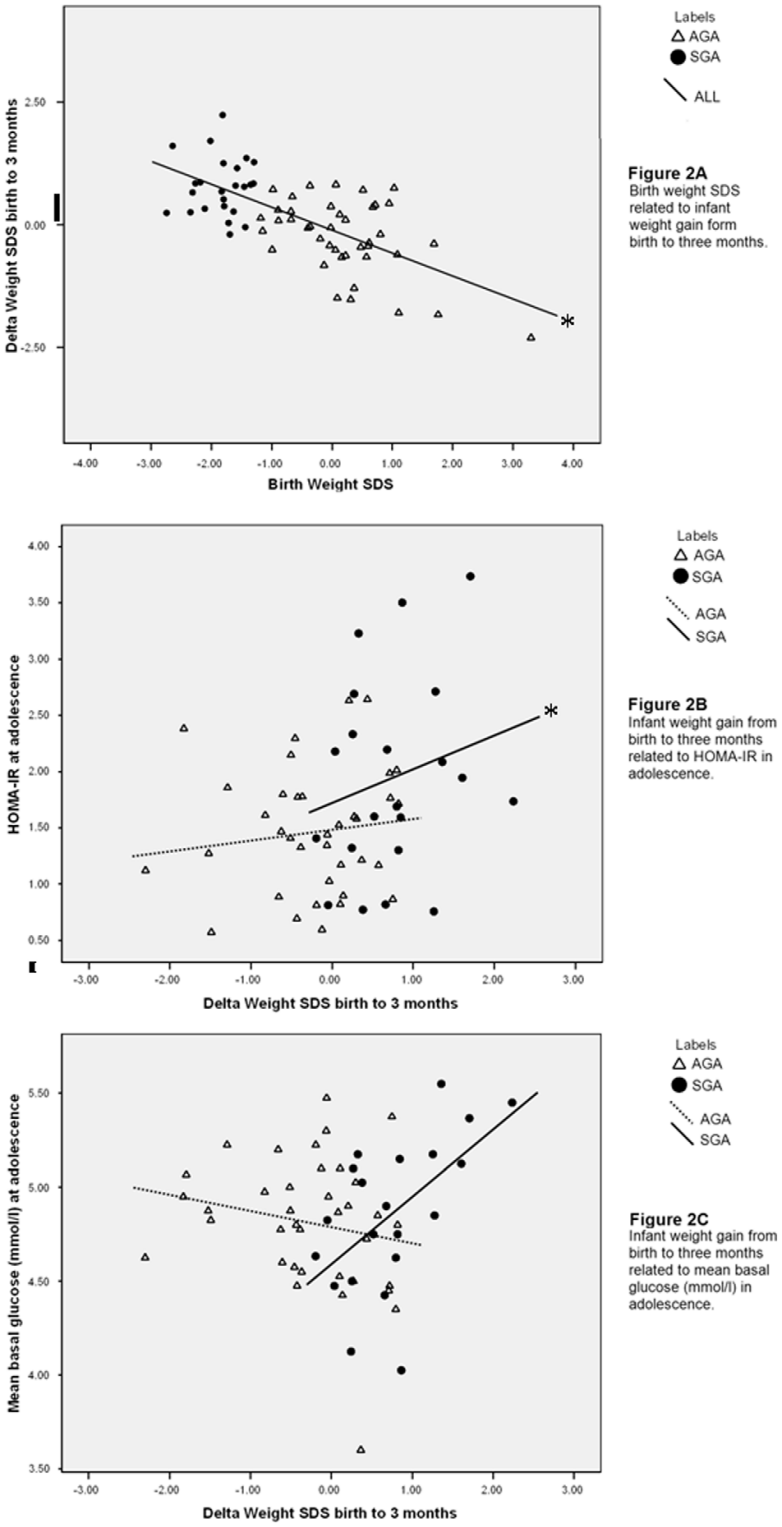
Correlations of infant weight gain with birth weight, HOMA-IR and plasma glucose. Correlations between infant weight gain from birth to 3 months and birth weight SDS (Panel A), HOMA-IR at adolescence (Panel B), and mean basal plasma glucose concentration at adolescence (Panel C) in individuals born appropriate for gestational age (AGA) and small for gestational age (SGA). *: *P*<0.005 for association.

Individuals in the SGA group had lower birth weight SDS (in agreement with the classification), and higher absolute early infant weight gain from birth to three months and to one year (Table 1). At one year of age, children born SGA were still considerably smaller than those born AGA (−1.28+/−0.94 SDS vs. −0.52+/−0.90). There was an inverse correlation between birth weight SDS and infant weight gain at both 0–3 months (r = −0.67, *P*<0.0001; Figure 2A) and 0–12 months (r = −0.67, p =  <0.0001). In adolescence, individuals born SGA were shorter, although slightly older at examination, compared with the AGA individuals (Table 1). There were no significant differences in measures of adiposity between the groups.

### Effects of early infant weight gain on markers of the metabolic syndrome

#### Effects in the entire group

In the entire group, ΔWSDS_birth to 3 months_ was positively associated with basal insulin concentration and HOMA-IR, as well as with plasma triglyceride, total cholesterol, LDL, and systolic blood pressure at adolescence (Table 2). Furthermore, we found a near-significant inverse association between ΔWSDSbirth to 3 months and insulin sensitivity (SI derived from IVGTT) and a near-significant positive association between ΔWSDS_birth to 3 months_ and fat mass (Table 2). Of the tested variables, ΔWSDS_birth to 1 year_ was only positively associated with HOMA-IR (B = 0.13; 95% CI: 0.000 to 0.27) (P = 0.05).

**Table 2:** Effects of early infant weight gain (ΔSDS_birth to 3 months_) on glucose metabolism, body composition, and cardiovascular risk factors at adolescence.

		ALL	AGA	SGA
		(n = 65)	(n = 41)	(n = 24)
**Glucose metabolism**	**N**			
Basal glucose (mmol/l)	58	0.01±0.05	−0.06±0.06	0.28±0.14
*K*_g_ (10^2^ min^−1^)	54	−0.15 [−0.36; 0.08]	−0.08 [−0.40; 0.29]	−0.34 [−0.78; 0.26]
Basal insulin (pmol/l)	59	7.33 [2.28; 13.0] ***	2.53 [−3.06; 9.09]	18.3 [0.65; 42.4] *
HOMA-IR	57	0.29 [0.10; 0.51] **	0.07 [−0.14; 0.30]	0.99 [0.27; 1.99] **
Insulin sensitivity (10^5^ min^−1^ per pmol l^−1^ min)	54	−0.07 [−0.12; 0.002]	−0.07 [−0.15; 0.04]	−0.10 [−0.20; 0.07]
First-phase insulin response (pmol l^−1^ min per mmol l^−1^)	54	2.04 [−4.45; 10.0]	4.65 [−5.39; 18.3]	−4.73 [−15.94; 14.1]
DI (10^5^ min−1)	54	−77.5 [−220; 104]	−23.9 [−258; 309]	−224 [−421; 110]
**Body composition**				
Lean body mass (kg) ^§§^	61	−0.85 [−1.90; 0.22]	−0.44 [−1.95; 1.12]	0.10 [−2.88; 3.18]
Fat mass (kg)^§§^	61	0.63 [−0.03; 1.32]	0.29 [−0.56; 1.23]	0.71 [−1.33; 3.13]
Trunk fat (%)	61	1.40±0.85	1.08±1.12	1.97±2.53
Waist/Hip ratio	65	0.01 [−0.01; 0.03]	0.00 [−0.02; 0.02]	0.00 [−0.07; 0.08]
**Lipid profile**				
Triacylglycerol (mmol/l)	60	0.15 [0.04; 0.28] **	0.07[−0.07; 0.24]	0.15 [−0.17; 0.60]
Cholesterol(mmol/l)	63	0.23 [0.01; 0.48] *	0.15 [−0.18; 0.50]	0.44 [−0.20; 1.21]
LDL-cholesterol (mmol/l)	62	0.25 [0.04; 0.48] *	0.24 [−0.08; 0.60]	0.22 [−0.29; 0.85]
HDL-cholesterol (mmol/l)	63	−0.03 [−0.12; 0.06]	−0.05 [−0.16; 0.07]	0.17 [−0.13; 0.54]
**Blood pressure**				
Systolic BP (mmHg)^§^	65	2.63±1.30*	−0.20±1.90	2.82±3.23
Diastolic BP (mmHg)^§^	65	1.65±1.02	1.14±1.49	1.14±2.90

Data are linear regression coefficients ± SE or [95% confidence limits]. For the purpose of tabulation the regression coefficients and confidence limits for log-transformed dependent variables were scaled using the regression coefficient for the untransformed variable. All regressions are adjusted for sex. ^§^Adjusted for sex, age and current height. ^§§^Adjusted for sex and current weight. ***P<0.001, **P<0.01, *P<0.05.

#### Effects in the SGA and AGA groups

General linear models showed differential effects of ΔWSDS_birth to 3 months_ in the SGA and AGA on both HOMA-IR (P for interaction = 0.03, Figure 2B) and mean basal glucose concentration (P for interaction = 0.04, Figure 2C). Similarly, the effect of ΔWSDS_birth to 1 year_ on HOMA-IR was significantly different in the SGA and AGA groups (P for interaction = 0.03). In the SGA group, the association of ΔWSDS_birth to 3 months_ with basal insulin levels and HOMA-IR was positive (Table 2 and Figure 2B). After adjustment for measures of adiposity in adolescence (BMI, waist-hip ratio, or trunk fat percentage), the association of early infant weight gain (ΔWSDS_birth to 3 months_) with HOMA-IR remained significant in the SGA group (P<0.05). ΔWSDS_birth to 1 year_ was also positively associated with HOMA-IR in the SGA group (*P* = 0.02). In the AGA group, early infant weight gain from birth to three months (Table 2) or to one year (data not shown) was not associated with any of the studied parameters.

#### Lifestyle and socioeconomic factors

To assess whether the observed differences between SGA and AGA in this study were due to differences in lifestyle factors, we compared physical activity, nutrition, breastfeeding, and smoking habits of the participants, and in addition we compared employment, education, health, physical activity and nutrition of the parents in the SGA and AGA groups with data on postnatal growth. However, there were no significant differences between the SGA and AGA group in regard to these parameters, or with regard to the response rate of the questionnaire (P>0.05 for all; data not shown).

## Discussion

In this prospective study we observed a relationship of early infant weight gain from birth to three months with glucose metabolism, lipid profile, fat mass and systolic blood pressure in adolescence. Interestingly, the associations to glucose metabolism were only apparent in adolescents born SGA, supporting that catch-up growth is adversely associated with disturbances of glucose metabolism later in life, while accelerated weight gain in infants born AGA does not seem to be particularly detrimental for glucose metabolism in adolescence. Our finding that early infant weight gain had different effects on glucose metabolism but similar effects on lipid profile and blood pressure among the SGA and AGA groups is novel, and support a growing awareness of differential trajectories and mechanistic pathways of the influence of pre- versus post-natal growth on the development of diabetes, stroke and coronary heart disease [12]. In other words, adverse effects occurring during different time points in pregnancy on a distinct metabolic outcome relevant to the metabolic syndrome may differ from adverse developmental programming effects occurring after birth and vice-versa. The exact causes and underlying mechanisms responsible for our findings of differential effects on glucose and lipid metabolism remain to be determined. However, epigenetic mechanisms including DNA methylations may be involved. For instance, the associations between early infant weight gain, HOMA-IR and basal insulin in adolescents born SGA could be explained by higher DNA methylation patterns of key regulators of glucose metabolism in those born SGA compared with those born AGA. A recent study from our group showed that young men born SGA had increased DNA methylation of the promoter region of the key metabolic regulator peroxisome proliferator-activated receptor gamma, coactivator 1alpha (PPARGC1A) influencing insulin action in muscle compared with matched controls born AGA [29]. Furthermore, the men born SGA had a much lower responsiveness of DNA methylation to the “diabetogenic” challenge of 5 days of overfeeding compared with men born AGA. This suggests that growth restriction early in life may lead to a less dynamic epigenetic flexibility when exposed to environmental challenges in adulthood. However, the field of epigenetics in developmental programming is currently at its infancy and much more studies are needed before it can be concluded whether DNA methylation plays any role in programming of human metabolism.

Other studies have shown adverse effects of accelerated early infant growth on glucose metabolism in infants and children born SGA. These findings include increased basal insulin levels associated with growth of weight between birth and one year [6] or three years [17] of age, and a positive association between linear growth and insulin secretion during IVGTT at six months [30] and at one year of age [6]. Leunissen *et al.* found that rapid growth during the first 3 months of life was associated with insulin resistance and markers of cardiovascular disease in young adults independent of birth weight SDS [31]. In contrast, some population-based studies have reported that low weight gain during infancy followed by accelerated weight gain during later childhood is associated with an increased risk of developing ischaemic heart disease, type 2 diabetes, and the metabolic syndrome [7]–[11]. Hence, the timing of rapid weight gain during infancy and childhood seems rather important for later development of health and disease. Our novel observation that rapid infant weight gain during the first three months of life causes adverse effects on glucose metabolism, lipid profile and hypertension among adolescents born SGA may have been missed in previous studies for a number of reasons. These include lack of correction for birth weight, less frequent and/or accurate determinations of weight during infancy and as well as differences in accuracy and standardizations of measurements during adolescence. Other factors such as secular trends and general living conditions during different time periods may influence the impact and timing of adverse developmental programming. Underfeeding of infants and young children was quite common in the beginning of the 20^th^ century, whereas overfeeding has become more common during recent years. Therefore, it may be speculated that in cohorts of people born in the 1930–1940's, the adverse metabolic programming could potentially occur at a later time during childhood than in the cohorts born in the 1980–1990's.

Adiposity seems to play a major role in the association between low birth weight, childhood growth and adult disease [16], [32]–[38]. Therefore, it is noteworthy that measures of adiposity in adolescence did not differ between the AGA and SGA groups, nor was accelerated growth during infancy associated with adiposity in adolescence in our study. This may be due to the relatively low number of individuals in our study, since other larger studies have found clear associations of birth weight and early weight gain with later obesity [36], [37]. The European Youth Heart Study [37] recently showed that higher birth weight was associated with higher fat mass index and waist circumference in 1,254 9 year-old children and 15 year-old adolescents. Also the ALSPAC study of more than 8,000 7-year-old children [36] provided evidence that birth weight, weight gain in the first year of life as well as catch-up growth and other risk factors in early life are associated with adiposity in childhood. In other studies risk of obesity has also been positively associated with weight gain in the first week of life [39], between birth and four months [40], between birth and two years of life [41], and between ages two and four years [42]. However, only few prospective studies until now have obtained precise recordings of early infant growth and detailed measurements of later metabolic health. One of them found that infant weight gain from birth to 6 months was positively associated with metabolic risk at 17 years of age [3]. No test was performed to assess whether the associations were similar across birth weight groups. However, after adjustment for birth weight SDS, the associations of early infant weight gain with lipids and blood pressure later in life remained statistically significant and the association to basal insulin was near-significant [3]. This supports an independent effect of early infancy weight gain with adverse cardio-metabolic features later in life. Also in our study, the associations between early infant weight gain and insulin resistance in the SGA group remained significant after adjustment for current BMI, fat mass or fat percentage, indicating that the effect of early infant weight gain on insulin resistance goes beyond an effect of predisposition to adiposity. This finding is in accordance with other studies where the effect of birth weight on insulin resistance and other metabolic risk factors in adolescence and adulthood also persisted after adjustment for current obesity degree [3], [43].

Previously, we showed in this cohort of adolescents that being born SGA was associated with a wide range of markers of the metabolic syndrome [18]. In the present analyses, we found that accelerated early infant weight gain from birth to three months was also associated with markers of the metabolic syndrome: increased basal insulin levels, HOMA-IR, adverse lipid profile, and hypertension. The extent to which the impact of growth during the first year of life was explained by growth during the first three months could not be assessed, because too few subjects had data on both variables of early infant weight gain (only 17 SGA subjects). Nevertheless, our results indicate that acceleration of early infant weight gain may aggravate the effect of low birth weight on glucose metabolism later in life, and that especially the first three months after birth represent a critical window. The notion of a critical window of developmental programming in humans shortly after birth is indeed supported by animal studies. Harder *et al.* infused daily doses of long-acting insulin or saline from the 8^th^ to the 11^th^ day of life in Wistar rats [44]. The animals receiving insulin infusion developed more obesity, insulin resistance and glucose intolerance in adulthood, illustrating that short-term insulin administration during a critical period of postnatal life has serious long-term consequences for glucose regulation in rats. In another study, the glucagon-like peptide-1-receptor agonist Exendin-4 was injected daily from day 1 to day 6 after birth in intrauterine growth-retarded rats [45]. Interestingly, this short-term infusion of Exendin-4 right after birth reversed the adverse consequences of fetal undernutrition on glucose metabolism later in life. Most strikingly, the rats treated neonatally with Exendin-4 showed improved glucose tolerance, normalized beta cell proliferation and even reduced mortality later in life compared to vehicle-treated intrauterine growth-retarded rats [45]. Taken together, the studies indicate that it is possible to induce as well as to protect against dysmetabolic traits by endocrine manipulations during a critical window right after birth. Our data suggest that similar mechanisms operate in humans and that the time period shortly after birth is a window of opportunity for prevention of later metabolic abnormalities. However, as already indicated above, there may be other critical time windows during gestation as well as during postnatal development where interventions may have similar or even larger effects on future health and disease.

In a previous publication [18], we showed that fetal growth in the last trimester does not have an effect on the metabolic phenotype in adolescence [18]. Hence, the most sensitive period in fetal life appears to be before the 3^rd^ trimester. Thus, paradoxically, growth in the first three months after birth seems to be more important than growth in the last three months before birth. This suggests that birth in itself opens a new window of sensitivity to metabolic programming, perhaps as an adaptive response to the change of environment. Efforts to alter the postnatal environment, i.e. to restrict early infant weight gain in SGA individuals, have hitherto not been recommended. Weight gain during the first months after birth is influenced both by the mode of feeding and the amount of energy eaten. A systematic review has shown that breastfeeding is associated with a reduced risk of type 2 diabetes later in life [46]. Unfortunately, the quality of the data on dietary intake during the first three months of life in our study did not allow an analysis of the potential effects of early feeding.

In conclusion, we found that accelerated growth during the first three months of life was associated with later metabolic disturbances – particularly of glucose metabolism – in individuals born SGA. Our findings thereby support the idea that the very early postnatal period is a critical window for individuals who have experienced a growth insult in fetal life.
